# Riceberry Rice Germination and UVB Radiation Enhance Protocatechuic Acid and Vanillic Acid to Reduce Cellular Oxidative Stress and Suppress B16F10 Melanogenesis Relating to F-Actin Rearrangement

**DOI:** 10.3390/plants12030484

**Published:** 2023-01-20

**Authors:** Parichaya Aimvijarn, Witchuda Payuhakrit, Nisamanee Charoenchon, Seiji Okada, Prasit Suwannalert

**Affiliations:** 1 Department of Pathobiology, Faculty of Science, Mahidol University, Bangkok 10400, Thailand; 2 Pathobiology Information and Learning Center, Department of Pathobiology, Faculty of Science, Mahidol University, Bangkok 10400, Thailand; 3 Division of Hematopoiesis, Joint Research Center for Human Retrovirus Infection, Graduate School of Medical Sciences, Kumamoto University, Kumamoto 860-0811, Japan

**Keywords:** riceberry rice, melanogenesis, F-actin, UVB radiation, germination and oxidative stress

## Abstract

Ultraviolet type B (UVB) radiation plays an important role in hyperpigmentation disorder, which induces cellular oxidative stress and causes abnormal melanin production and secretion. The stress condition plays an essential role in actin polymerization relating to F-actin rearrangement and forms dendrite to send melanin pigment to the uppermost layer of the skin. Phenolic compounds are secondary metabolites that mainly synthesize under stress conditions to protect plants from harmful environments and have been reported as effective agents in anti-oxidant and anti-melanogenesis. However, the influence of phenolic compounds on F-actin rearrangement-associated dendrite formation has not been studied so far. Hence, this study aimed to investigate the enhancing phytophenolic targets in riceberry rice (*Oryza sativa* L.) germination and UVB radiation (RR-GR) to suppress melanogenesis relating to F-rearrangement. As a result, the RR-GR had the potential to enhance phenolic acids such as protocatechuic and vanillic acid, which have been proven to possess anti-oxidant activity and anti-tyrosinase properties. Riceberry rice’s modification showed the potential to reduce cellular oxidative stress and suppress B16F10 melanogenesis relating to F-actin rearrangement that is associated with dendrite formation.

## 1. Introduction

Ultraviolet type B (UVB) radiation is the extrinsic factor penetrating through the epidermis layer and absorbing melanocytes. The melanocytes are naturally located in the skin’s uppermost layer and lead to melanin production [1]. This radiation plays an important role in promoting the formation of free radicals and causes cellular oxidative stress [2]. Stress condition is the factor that leads to aggressive melanogenesis associated with tyrosinase enzyme activity [3] and promotes dendrite formation to send melanin to the uppermost layer of the skin [4]. The dendrite formation involved with Filamentous-actin (F-actin) rearrangement is an essential molecule in cell morphology change and melanin pigment transportation [5,6]. Phenolic compounds have been reported to be essential nutritional ingredients by protecting humans from stress conditions and with their biological functions known as anti-oxidant activity [7]. Moreover, they can suppress melanogenesis by acting as a tyrosinase enzyme inhibitor. These compounds play an important role as competitive inhibitors acting as copper chelators or compounds structurally mimicking tyrosinase substrates [8]. Phenolic compounds such as protocatechuic acid, vanillic acid, caffeic acid, and ferulic acid are mostly found in pigmented rice as secondary metabolites [9]. The purple pigments have been reported to act as anti-oxidant agents related to the number of OH groups in the phytophenolic structure, scavenging free radicals [10]. Germination can promote secondary metabolites by increasing the oxidative forms of pentose phosphate and aromatic acid, which can convert into phenolic compounds [11] and UVB radiation; abiotic factors promote self-defense mechanisms. UVB radiation can also damage and induce ROS accumulation in plant cells [12]. Therefore, plants have developed themselves to be protected from environmental stress conditions by synthesizing effective anti-oxidants such as phenolic compounds that act as ROS scavengers and photoprotection from the UVB radiation [13,14]. However, the combination of these processes to enhance the ability of phytophenolic targets has not been studied. So, Ultraviolet type B (UVB) radiation induces ROS accumulation and causes cellular oxidative stress. The stress condition increases abnormal melanogenesis and promotes dendrite formation leading to hyperpigmentation disorder. On the other hand, phenolic compounds are well-recognized anti-oxidants and tyrosinase enzyme inhibitors that play an essential role in anti-melanogenesis, but F-actin-related dendrite formation is unknown. This study aims to enhance phenolic compounds in riceberry rice by passing through germination and UVB radiation. The increasing phenolic targets, protocatechuic acid, and vanillic acid may play a crucial role in anti-oxidant, anti-tyrosinase, and anti-melanin production-related F-actin rearrangement.

## 2. Materials and Methods

### 2.1. Riceberry Rice Sample Prepration

The riceberry rice (RR), which is dark purple and is the local rice of Thailand, is cross-bred between Thai Hom Nil and Jasmine rice. Organic RR was obtained from Roi Et Province, Thailand (Organic farm code: CU84886). The rice was devised into three conditions which are riceberry rice (RR), riceberry rice with germination (RR-G), and riceberry rice with germination and radiation (RR-GR). First, rice was soaked in distilled water for 5–10 min. Then, RR-G and RR-GR conditions were germinated for 6 h. After that, the RR-GR condition was radiated with Ultraviolet type B (UVB) at 0.5 J/cm^2^ for 10 min and kept at −20 °C with a modified method [15]. The extraction process was modified from the previous study [16]. In brief, rice samples were blended and mixed with 95% ethanol analytical grade under reflux for 4 h. The soluble part was dried in a rotary vacuum evaporator (Buchi rotavapor R-200, Switzerland). The residues were suspended in distilled water (DW) and dried in a freeze-drier (Supermodulyo-230, Singapore). The extract samples were collected and frozen at −80 °C. For cellular investigation, the samples were dissolved in Dulbecco’s Modified Eagle’s medium (DMEM) (Sigma-Aldrich, Singapore) and filtered with 0.45 μm polytetrafluoroethylene (PTFE) filter nylon before use in the experiment.

### 2.2. Radical Scavenging Activity by 2,2-Diphenyl-1-picryl-hydrazyl-hydrate (DPPH) Assay

The anti-oxidant property was investigated by a modified DPPH method [17]. The working solution of DPPH was freshly prepared by being dissolved and diluted with 95% ethanol. The absorbance was measured at 530 nm by use of a UV-2650 spectrophotometer (UV-2650, Labomed, Los Angeles, CA, USA). Samples were mixed with a DPPH solution. The mixtures were measured at 530 nm against the blank by a spectrophotometer (UV-2650, Labomed, Los Angeles, CA, USA). The radical scavenging activity of each sample was measured as a decrease in the absorbance of DPPH. The total anti-oxidant activity in the DPPH test was expressed as a mg vitamin C equivalents/g sample.

### 2.3. Ferric Reducing Anti-Oxidant Power (FRAP) Assay

The reduction capacity of samples with a modified method [18]. First, the working FRAP reagent was prepared. Then, in dark conditions, the samples were mixed with a working FRAP reagent in 96-well plates for 15 min. Readings of the blue-colored product (ferrous tripyridyltriazine complex) were measured at 595 nm using an automated microplate reader (1420 Victor 2, Wallac, ID, USA). Iron (III) sulfate heptahydrate (FeSO_4_.7H_2_O) was used as a standard, and the optical density (OD) of the sample and standard was expressed as a linear graph. The reduction activity was expressed as mmol FeSO_4_.7H_2_O equivalents/g sample.

### 2.4. Total Phenolic Content Assay

Total phenolic content was determined with a Folin-Denis reagent [19]. Twenty microliters of the sample were mixed with 1580 µL of distilled water and then combined with 100 µL of the Folin-Denis reagent. The mixture was incubated with 300 µL of 7.5% of sodium bicarbonate for 30 min at room temperature in dark conditions. Next was the absorbance of 765 nm using a UV-2650 spectrophotometer (Labomed, Los Angeles, CA, USA). Gallic acid was used as a standard. The OD of the sample and standard were determined to be the total phenolic content expressed as a value of mg gallic acid equivalents/g sample.

### 2.5. Tyrosinase Activity (In Vitro Mushroom Tyrosinase Activity)

The mushroom tyrosinase (SLBZ0022) (Sigma-Aldrich, Singapore) was studied under some modifications [20]. First, 100 µL of phosphate buffer (0.1 M, pH6.8) was incubated with 50 µL of samples and 50 µL of mushroom tyrosinase at room temperature for 10 min in 96-well plates. Then, mixture samples were mixed with 20 µL of 1 mM 3,4-dihydroxyphenylalanine (L-DOPA) (Sigma-Aldrich, Singapore) at 25 °C for 15 min. The absorbance of dopachrome was measured at 492 nm using a microplate reader (OPTIMax, Tunable). Kojic acid was used as standard. The results were expressed as the percentage of tyrosinase enzyme activity.

### 2.6. High-Performance Liquid Chromatography (HPLC)

To identify the phenolic compounds in the investigated samples, the modified HPLC method was applied [21]. The standards of this study are, 1: protocatechuic acid (PA), 2: vanillic acid (VA), 3: caffeic acid (CA) 4: ferulic acid (FA) with ≥99.0% (HPLC) from Sigma-Aldrich. First, standards and samples were eluted by absolute acetonitrile solution (A) and 0.1% trifluoroacetic acid solution in distilled water (B) under the gradient (Solvent A: Solvent B) at 5:95%, 10:90%, 15:85%, 25:72%, 50:50%, and 80:20% with a respective time period of 0–5, 5–20, 20–35, 35–40, and 40–50 min. The flow rate was controlled at 0.8 mL/min by an ACE^®^ C18 column (Advanced Chromatography Technologies, Aberdeen, Scotland) (250 mm × 4.6 mm; 5 µm) at a temperature of 40 °C. The fingerprints of phytophenolics were detected with a UV detector at 280 nm. The commercial standards were from Sigma-Aldrich, Singapore, to design for fingerprint identification.

### 2.7. Cell Culture

B16 mouse melanoma cells (B16F10 cells) (ATCC Number CRL-6475TM, Virginia, USA) were routinely cultured in Dulbecco’s modified Eagle’s medium (DMEM). The media were supplemented with 10% heat-inactivated fetal bovine serum (FBS), 1% non-essential amino acid, 1% L-glutamine, 1% penicillin-streptomycin, and 3.7 mg/mL of NaHCO_3_. Cells were maintained at 37 °C in a humidified atmosphere that was supplied with a 5% CO_2_ incubator.

### 2.8. Cell Viability by 3-(4,5-Dimethyl-2-yl)-2,5-Diphenyltetrazolium Bromide (MTT) Assay

The MTT assay evaluated was used to investigate cell viability by cleavage yellow tetrazolium salt (4,5-dimethylthiazol-2-yl)-2,5-diphenyltetrazolium bromide (MTT) into purple formazan by metabolically active cells. The increasing of living cells leads to a stronger purple color formation [22]. Cells were seeded in the 96-well plates and maintained for 24 h. After the incubation time, cells were treated with various concentrations of UVB and samples. Then, the treated cells were incubated within 24 h. After the incubation time, cells were removed from the old media and washed with PBS buffer, pH 7.4. To detect the cell viability, treated cells were then treated with MTT solution and incubated for 4 h. The results were investigated by solubilizing the purple formazan with dimethyl sulfoxide (DMSO). The color was read using an automatic microplate reader (1420 Victor 2, Wallac, ID, USA) at a wavelength of 570 nm. The results were expressed as the percentage of viable cells (% cell viability).

### 2.9. Cellular Oxidative Stress by Dichloro-dihydro-fluorescein diacetate (DCFH-DA) Assay

Cellular oxidative stress was detected with DCFH-DA in a modified method [20]. DCFH-DA is cleaved in intracellular cells by nonspecific intracellular esterase and turns to highly fluorescent 2,7-dichlorofluorescein (DCF) upon oxidation by free radicals. B16F10 cells were seeded in 96-well plates and incubated at 37 °C, 5% CO_2_ for 24 h. Cells were radiated with UVB and then treated with various concentrations of the sample for 24 h. Cultured cells were removed from the conditioned medium. DCFH-DA mediums were added and incubated at 37 °C, 5% CO_2_ for 1 h. DCF fluorescence intensity was immediately assessed for cellular oxidative stress at excitation/emission wavelengths of 485/535 nm by using a fluorescence microplate reader (1420 Victor 2, Wallac, ID, USA). N-acetyl cysteine (NAC) was used as a positive anti-oxidant with 100% control for untreated cells. The result was expressed as the percentage of cellular oxidative stress (% cellular oxidation).

### 2.10. Melanin Containing Cell

The melanin content in B16F10 cells was determined as previously described [21]. Cells were seeded into 6-well plates at 1.0 × 10^5^ cell/well and incubated for 24 h. Then, we let the cells attach to the plate. Then, the cells were pretreated with UVB radiation at 0.5 J/cm^2^ and immediately treated with various concentrations of the samples. The treated cells were incubated for 48 h with daily treatment. Treated cells were maintained at 37 °C in a humidified atmosphere that supplied a 5% CO_2_ incubator. After the incubation time, old media were removed and washed with PBS buffer, pH 7.4 three times. Then, the Fontana-Masson staining was conducted using a Fontana-Masson Stain Kit (Bio Opitical, Italy) according to the manufacturer’s instructions. The 1000 B16F10 cells were counted and the melanin-containing cells were recorded under a light microscope at 100× magnification. The melanin-containing cells were expressed as a percentage of the number of melanin-containing cells compared with the control as 100%. Kojic acid at 1 mM was used as a positive melanin inhibitor. This assay was carried out in triplicate.

### 2.11. Melanin Content and Secretion Assay

The melanin content in B16F10 cells was measured (tested) by use of modified methodology from previous studies [23]. Cells were seeded into 24-well plates and incubated for 24 h. Next, cells were pretreated with UVB radiation and treated with various concentrations of samples, then incubated for 48 h. At first, to investigate the melanin secretion, B16F10 cells were determined in a culture medium at 405 nm. Then, the melanin content B16F10 cells were washed with phosphate buffered saline (PBS) pH 7.4, lysed with 100 µL of 1 N NaOH, 1% TritonX-100, 1 mM phenylmethanesulfonyl fluoride (PMSF), and incubated at 80 °C for 2 h. The lysate was measured by using a microplate reader (1420 Victor 2, Wallac, ID, USA) at 405 nm. Kojic acid was used as a positive melanin inhibitor. The melanin content was expressed as a percentage of control, 100% of control.

### 2.12. Cell Morphology and F-Actin Cytoskeleton Labelling

To study morphology by F-actin, the labeling of B16F10 was observed by a fluorescence microscope with some modifications [24]. B16F10 cells were cultured in 30 mm culture plates in standard culture conditions for 24 h. Then, cells were radiated with UVB then treated with various concentrations of sample for 48 h with a daily treatment. For labeling, cells were permeabilized with 0.1% triton in PBS for 5 min. To investigate the cell morphology, cells were fixed with cold methanol for 30 min and washed with cold PBS pH 7.1. Then, treated cells were stained with a primary antibody against F-actin 5 µg/mL for 1 h and washed with cold PBS pH 7.1, and next, the secondary antibody Fluor^®^ 488 1:250 and washed with cold PBS pH 7.1 three times. Finally, the nucleus of the cells was stained with Hoecsht 33258 (1:1000). Cells were maintained in a mounting medium (30% Glycerol). The number of dendrite formation were calculated and presented in a bar graph. Morphological appearances of B16F10 cells were scored as 1+, 2+, 3+, and 4+ according to scoring criteria from a previous study [14], as shown Table 1 below:

### 2.13. Statistical Analysis

All results were presented as mean ± standard deviation (mean ± SD). The *t*-test was used to compare the significant differences between the treated and untreated cells. The post hoc test part of the one-way analysis of variance (ANOVA) was used for significant differences between treated groups. The significance was considered at *p* ≤ 0.05 with SPSS version 16-computer software.

## 3. Results

### 3.1. Ultraviolet Type B (UVB) Radiation on Cellular Melanin Content and Dendrite Formation

**Figure 1:**
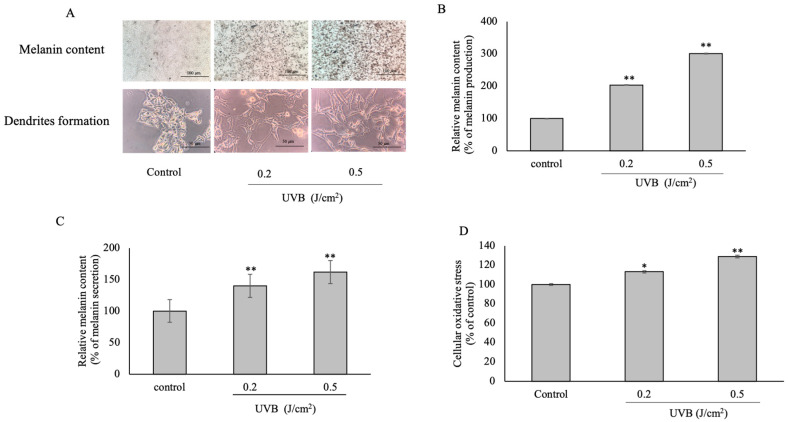
The effect of UVB on B16F10 cells at different concentrations (J/cm^2^), Pictures of melanin content, and dendrite formation of UVB induced cells (**A**) The melanin production (**B**) and secretion (**C**) are related to melanin content and dendrite formation. Results were expressed in a bar graph as the percentage of melanin production and secretion. Cellular oxidative stress was present as a bar graph (**D**). The statistical significance of differences of each group was calculated by mean ± SD with the control (100%). *, ** Statistical significance at *p* ≤ 0.05 and *p* ≤ 0.001, respectively.

In this study, the non-toxicity dose of UVB was up to 0.5 J/cm^2^. The aggressive cellular melanin pigment and dendrite formation after being induced by UVB were investigated and pictured under an inverted microscope (Figure 1A). Cellular melanin production related to melanin pigment accumulation also showed a significant increase at 205 ± 1.227% and 301 ± 0.965% at *p* ≤ 0.001 (Figure 1B), as well as melanin secretion associated with dendrite formation, which showed 140 ± 1.275% and 162 ± 0.275% at *p* ≤ 0.001 (Figure 1C). In addition, cellular oxidative stress was dramatically increased at 113 ± 1.131% and 129 ± 1.289% compared with the control (100%) (Figure 1D). Hence, UVB plays a critical stimulant factor in melanogenesis-associated dendrite formation and cellular oxidative stress.

### 3.2. Germination and Ultraviolet Type B (UVB) Radiation on Riceberry Rice-Related Anti-Oxidant Activity and Total Phenolic Content

Germination and UVB radiation were used to enhance phytophenolics-associated anti-oxidant activity in riceberry rice. Results found that riceberry rice with germination and radiation (RR-GR) showed the highest potential of anti-oxidant activity in both DPPH and FRAP assays at 1.31 ± 1.420 mg vitamin C/g sample and at 5.468 ± 0.143 M FeSO_4_·7H_2_O/g sample, respectively, when compared with riceberry rice (RR) (1.24 ± 0.770 mg vit C/g sample and 4.862 ± 0.091 M FeSO_4_·7H_2_O/g sample) at *p* ≤ 0.05 and *p* ≤ 0.001, respectively. In addition, the total phenolic content of RR-GR significantly increased at 1.412 ± 0.039 g gallic acid/g sample compared with riceberry rice (RR) (1.222 ± 0.031 g gallic acid/g sample)at *p* ≤ 0.001. Thus, the RR-GR extract showed the highest anti-oxidant activity and increased phenolic compound accumulation (Table 2).

### 3.3. The Extracts of Riceberry Rice (RR), Riceberry Rice with Germination (RR-G) and Riceberry Rice with Germination and Radiation (RR-GR) on Mushroom Tyrosinase Activity

**Figure 2:**
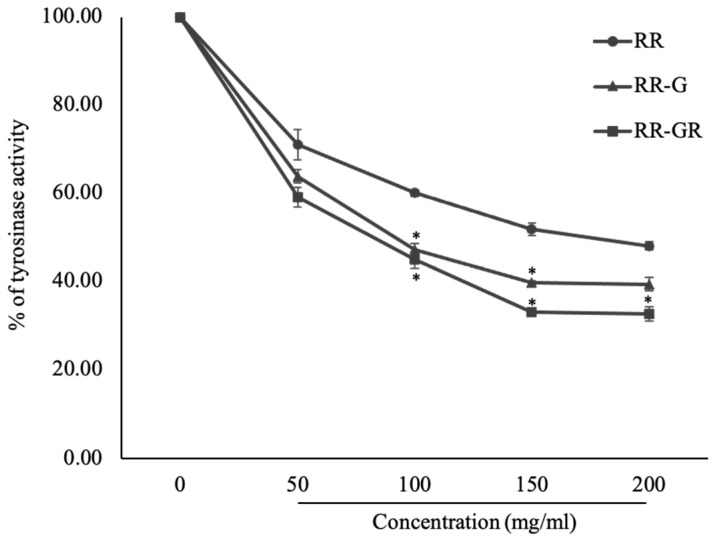
The suppression of tyrosinase enzyme activity of riceberry rice (RR), riceberry rice with germination (RR-G), and riceberry rice with germination and radiation (RR-GR) were investigated by mushroom tyrosinase assay. * Statistical significance at *p* < 0.05.

Tyrosinase activity related to melanogenesis was used to investigate the extracts’ ability. The results found that the RR-GR extract was in a significant decrease in tyrosinase enzyme activity from 100% to 32.70 ± 1.640% while RR-G at 39.41 ± 1.490% when compared with the RR sample (48.06 ± 0.920%) at *p* ≤ 0.05 in a dose-dependent manner (Figure 2). Hence, the RR-GR extract plays an important role in suppressing tyrosinase enzyme activity related to melanogenesis.

### 3.4. Phytophenolic Fingerprint of Riceberry Rice (RR), Riceberry Rice with Germination (RR-G) and Riceberry Rice with Germination and Radiation (RR-GR)

**Figure 3:**
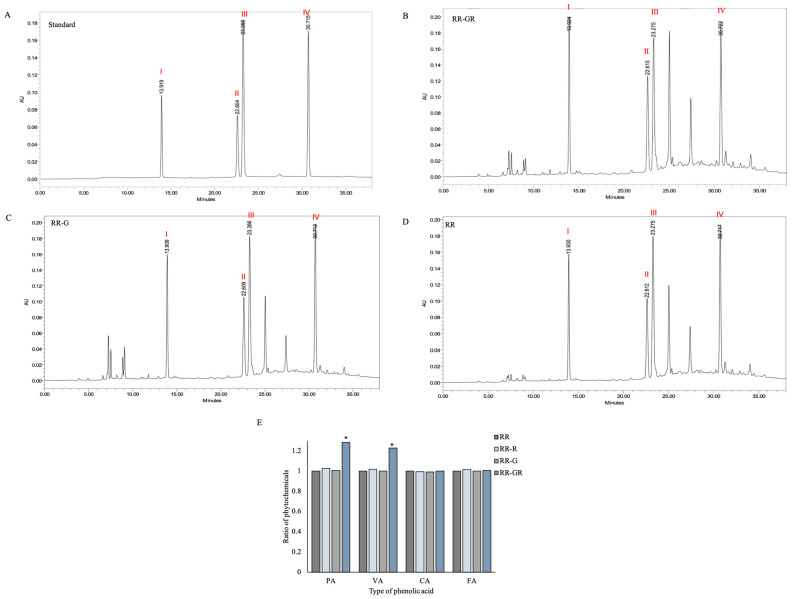
HPLC chromatogram of standard (**A**), riceberry rice with germination and radiation (RR-GR) (**B**), riceberry rice with germination (RR-G) (**C**), and riceberry rice (RR) (**D**). Percentage of area was presented in bar graph (**E**). The number represented in each type of phytochemicals I: protocatechuic acid (PA), II: vanillic acid (VA), III: caffeic acid (CA), IV: ferulic acid (FA). * Statistical significance at *p* < 0.05.

A phytophenolic fingerprint was used to study the predominant peak of riceberry rice (RR), riceberry rice with germination (RR-G), and riceberry rice with germination and radiation (RR-GR). The phytophenolic peaks were identified compared with standards as protocatechuic acid (I), vanillic acid (II), caffeic acid (III), and ferulic acid (IV) (Figure 3A). Results were investigated under the increasing ratio of targeted phytophenolics. The experiment found that the RR-GR extract (Figure 3B) showed a significant increase in protocatechuic acid, with an increasing ratio about 0.28 higher than RR-G (Figure 3C) and RR (Figure 3D), as well as in Vanillic acid, with the increasing ratio being about 0.23 higher than RR-G (Figure 3C) and RR (Figure 3D). Caffeic acid and ferulic acid did not show any significant differences. The increasing ratio is presented as a bar graph (Figure 3E). So, germination and UVB radiation play an essential role in enhancing phenolic accumulation. Hence, the combination of germination and UVB radiation processes on riceberry rice, RR-GR, showed a significant accumulation of phenolic compounds in a group of protocatechuic acid and vanillic acid, which also presented high antioxidant activity and suppressed tyrosinase enzyme activity. So, the RR-GR extract was a candidate to study in the cellular experiment.

### 3.5. Effect of Riceberry Rice with Germination and Radiation (RR-GR) on Cellular Oxidation in UVB-Induced Cells

**Figure 4:**
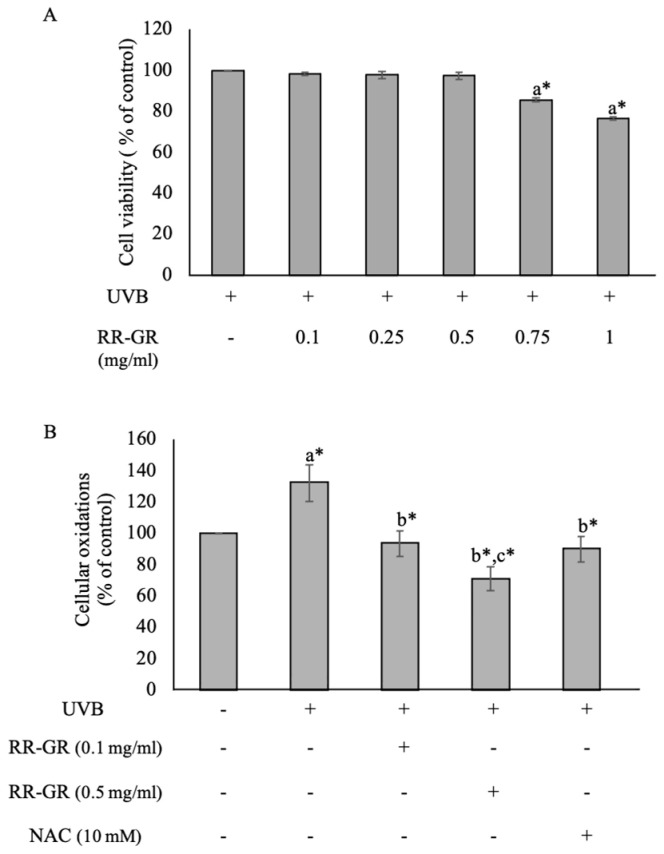
Cell viability was observed under MTT assay (**A**). Cellular oxidant was determined by the DCFH-DA method (**B**). N-acetylcysteine (NAC) was used as a positive control. The statistical significance of differences in each group was calculated by mean ± SD with the control (100%). ^a^ represents statistical significance compared with control. ^b^ represents statistical significance between radiated and treated cells. ^c^ represents statistical significance between treated cells. * Statistical significance at *p* ≤ 0.001.

In this study, the non-toxicity dose of the extract was used at 0.1 and 0.5 mg/mL, investigated by MTT assay (Figure 4A). The UVB radiation at 0.5 J/cm^2^ as a non-toxicity dose was exposed to cells before being treated with the RR-GR extract. The results showed that cellular oxidative stress was significantly increased in the radiation group with 132.23 ± 11.510% compared with the control (100%) at *p* ≤ 0.001. In contrast, RR-GR treated cells at 0.1 and 0.5 mg/mL were presented in dramatically reduced oxidative stress to 93.63 ± 8.240% and 71.05 ± 7.600% at *p* ≤ 0.001 in dose-dependent manners compared with the radiation group (132.23 ± 11.510%) (Figure 4B). NAC was used as a positive control. Thus, the RR-GR extract had a crucial role in suppressing cellular oxidative stress after exposure to UVB radiation.

### 3.6. Effect of Riceberry Rice with Germination and Radiation (RR-GR) on Melanin Production and Secretion in UVB-Induced Cells

**Figure 5:**
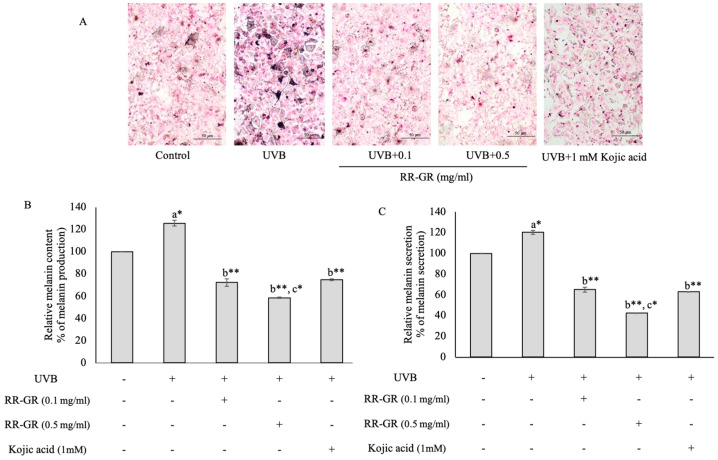
Melanin pigmentation and dendrite formation were determined by the Masson-Fontana assay. Control was present as untreated cells (**A**) The results of melanin production (**B**) and secretion (**C**) are present in the bar graph. The statistical significance of differences in each group was calculated by mean ± SD with the control (100%). ^a^ represents statistical significance compared with control. ^b^ represents statistical significance between radiated and treated cells. ^c^ represents statistical significance between treated cells. *, ** Statistical significance at *p* < 0.05 and *p* ≤ 0.001, respectively.

The pictured melanin pigment and dendrite formation were observed by Masson-Fontana assay. The UVB radiation on B16F10 cells significantly increased melanin accumulation and dendrite formation compared with the control (not UVB-treated). Whereas RR-GR treated at 0.1 and 0.5 mg/mL on UVB-induced cells were dramatically reduced in a dose-dependent manner compared with cells treated with UVB alone (Figure 5A). Kojic acid was used as a positive control. Cellular melanin production was investigated by melanin content assay. The results found that the radiation group showed a rapid increase at 125 ± 2.615% when compared with the control (100%), but when treated with RR-GR at 0.1 and 0.5 mg/mL, it reduced to 72.34 ± 3.389% and 58.527 ± 0.641% in a dose-dependent manner (Figure 5B). The dendrite formation related to melanin secretion was observed in cell culture media. In UVB-induced cells, it was shown at 120 ± 1.886% when compared with the control (100%). Surprisingly, after being treated with RR-GR at 0.1 and 0.5 mg/mL, UVB-induced cells were given a significantly reduced melanin secretion to 64.920 ± 2.190% and 42.471 ± 0.127% at *p* ≤ 0.001 in a dose-dependent manner (Figure 5C). Hence, the RR-GR extract was significantly suppressed in melanin production and secretion which was associated with the dendrite for;mation.

### 3.7. Effect of Ricebery Rice with Germination and Radiation (RR-GR) on Cellular Morphology Changes Related to F-Actin Rearrangement

**Figure 6:**
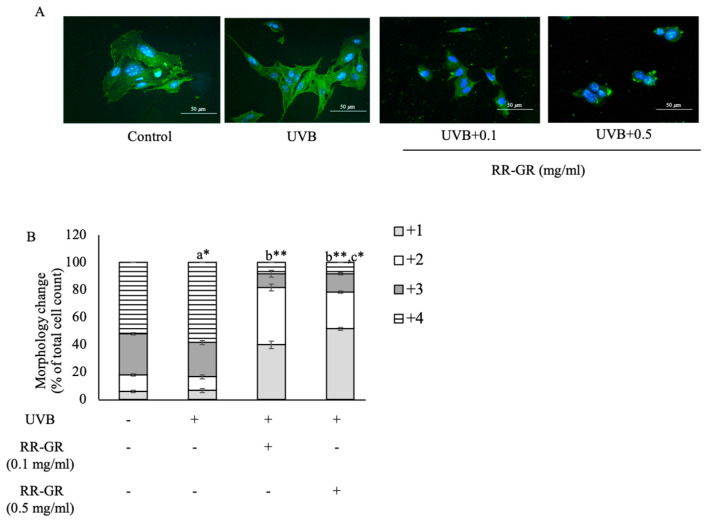
Morphology change-related F-actin rearrangement; the scale bar was at 50 µm. Control was present as untreated cells. (Blue: nucleus, green: F-actin rearrangement) (**A**). The morphology was graded as +1, +2, +3, and +4 from the total of 100 cells. The results were expressed as a percent cell morphology (**B**). ^a^ represents statistical significance compared with control. ^b^ represents statistical significance between radiated and treated cells. ^c^ represents statistical significance between treated cells. *, ** Statistical significance at *p* ≤ 0.05 and *p* ≤ 0.001, respectively.

The morphology of B16F10 cells was observed under the fluorescence microscope. First, the dendrite formation was invested followed by the localization of F-actin. This study was investigated under the F-actin rearrangement. In UVB-induced cells, dendrite formation-related F-actin rearrangement was presented higher than in the control (untreated cells), and RR-GR treated cells. Moreover, RR-GR treated cells at 0.1, and 0.5 mg/mL had significantly reduced dendrite formation in a dose-dependent manner (Figure 6A). F-actin arrangement-related dendrite formation was scored low to high at 1+, 2+, 3+ and 4+, respectively, and presented in the bar graph. UVB-induced cells were shown in the highest population of cells in score number 4+ at 57 ± 1.000%. In contrast, RR-GR treated cells at 0.1 and 0.5 mg/mL were at 7 ± 1.732% and 6 ± 1.211% in a dose-dependent manner. However, RR-GR treated cells at 0.1 and 0.5 mg/mL were mainly shown in the lowest dendrite formation; the score was 1+, at 38 ± 2.640% and 52 ± 1.000% in a dose-dependent manner (Figure 6B).

## 4. Discussion

Ultraviolet type B (UVB) radiation is a critical factor in inducing abnormal melanogenesis and dendrite formation in B16F10 cells [25]. The previous study supports that UVB radiation is a factor that promotes cellular oxidative stress. The overproduction of stress inside the cell is involved with the polymerization of F-actin rearrangement related to dendrite formation [26] and causes morphology change [5]. Moreover, UVB exposure is a considerable problem that causes hyperpigmentation and skin disorders [27]. B16F10 cells were exposed to UVB and showed high melanin production and secretion related to dendrite formation. This experiment was interested in a natural product that mainly contains phenolic compounds to reduce melanogenesis [28]. They show the unique structure of the hydroxyl group. This hydroxy group acts as competitive inhibitors [29] that bind to the tyrosinase enzyme, which plays a role in tyrosinase enzyme conformation change and suppresses the melanogenesis mechanism [30]. Moreover, pigmented rice mainly contained phytophenolics related to the number of OH groups in the phytophenolic structure, scavenging free radicals [10]. In addition, it shows the ability to act as an antioxidant to reduce oxidative stress inside cells [31] and to enhance the accumulation of phenolic compounds in riceberry rice containing abundant phytochemicals. Germination and UVB radiation processes were used as a modified method to enhance phytophenolic targets. Followed by the result, the RR-GR extract showed a significant increase in antioxidant activity and predominantly enhanced protocatechuic acid and vanillic acid. According to a previous study, seed germination is involved in converting seed material such as oxidative pentose phosphate, glycolytic, and aromatic amino acid into the phenolic compound [32], together with UVB radiation, which plays an important role to develop a defense mechanism through the synthesis of secondary metabolites such as phenolic compounds to prevent plants from harmful environmental stress [33,34].

Hence, RR-GR was the best condition to study the effectiveness of B16F10 melanin-producing cells. As mentioned in the previous study, the stress condition promotes melanogenesis and is also involved in the actin polymerization of stress fibers related to F-actin rearrangement to promote dendrite formation in the cells [35,36]. Therefore, after being treated with RR-GR extract on UVB-induced cells, it was shown to reduce cellular oxidative stress and suppress melanogenesis according to decrease the tyrosinase activity. The RR-GR extract’s ability to reduce cellular oxidation was involved in decreased F-actin rearrangement, which presents a low number of cell dendrite formation. Thus, germination and UVB radiation were enhancing phenolic compounds in riceberry rice and showed the effectiveness of suppressing melanogenesis and reducing F-actin rearrangement associated with cellular oxidative stress.

## 5. Conclusions

Phytophenolic targets, protocatechuic acid, and vanillic acid in riceberry rice were enhanced through germination and UVB radiation processes. Riceberry rice’s modification showed high anti-oxidant and anti-tyrosinase properties together with the potential to reduce cellular oxidative stress and suppress B16F10 melanogenesis relating to F-actin rearrangement associated with dendrite formation.

## Figures and Tables

**Table 1:** The scoring criteria of morphology appearance in B16F10 cells.

Criteria	Score
1. No dendrite shape and cell size at 1–50 μm	+1
2. Dendrite shape with bi-polar and cell size at 1–50 μm	+2
3. Dendrite shape with multi-polar and cell size larger than 51 μm	+3
4. Dendrite shape with multi-polar, more spindle shape and cell size larger than 51 μm	+4

**Table 2:** Antioxidant capacity and total phenolic content were represented in the results of riceberry rice (RR), riceberry rice with germination (RR-G), and riceberry rice with germination and radiation (RR-GR).

Conditions	Antioxidant Capacity		Total Phenolic Content
	DPPH (mg vit C/g Sample)	FRAP mmol FeSO_4_·7H_2_O/g Sample	Folin-Denis (mg Gallic Acid/g Sample)
RR	1.24 ± 0.770	4.862 ± 0.091	1.222 ± 0.031
RR-G	1.21 ± 1.400	5.449 ± 0.109 **	1.372 ± 0.064 **
RR-GR	1.31 ± 1.420 *	5.468 ± 0.143 **	1.412 ± 0.039 **

*, ** Statistical significance at *p* ≤ 0.05 and *p* ≤ 0.001, respectively.

## Data Availability

Data sharing not applicable.
